# Shape-controlled synthesis and influence of W doping and oxygen nonstoichiometry on the phase transition of VO_2_

**DOI:** 10.1038/srep14087

**Published:** 2015-09-16

**Authors:** Ru Chen, Lei Miao, Chengyan Liu, Jianhua Zhou, Haoliang Cheng, Toru Asaka, Yuji Iwamoto, Sakae Tanemura

**Affiliations:** 1 Key Laboratory of Renewable Energy, Guangzhou Institute of Energy Conversion, Chinese Academy of Sciences, Guangzhou 510640, P. R. China; 2 University of Chinese Academy of Sciences, Beijing 100049, P. R. China; 3 Guangxi Key Laboratory of Information Material, Guangxi Collaborative Innovation Center of Structure and Property for New Energy and Materials, School of Material Science and Engineering, Guilin University of Electronic Technology, Guilin, 541004, P. R. China; 4 Department of Frontier Materials, Graduate School of Engineering, Nagoya Institute of Technology, Gokiso-cho, Showa-ku, Nagoya, 466-8555, Japan

## Abstract

Monoclinic VO_2_(M) in nanostructure is a prototype material for interpreting correlation effects in solids with fully reversible phase transition and for the advanced applications to smart devices. Here, we report a facile one-step hydrothermal method for the controlled growth of single crystalline VO_2_(M/R) nanorods. Through tuning the hydrothermal temperature, duration of the hydrothermal time and W-doped level, single crystalline VO_2_(M/R) nanorods with controlled aspect ratio can be synthesized in large quantities, and the crucial parameter for the shape-controlled synthesis is the W-doped content. The dopant greatly promotes the preferential growth of (110) to form pure phase VO_2_(R) nanorods with high aspect ratio for the W-doped level = 2.0 at% sample. The shape-controlled process of VO_2_(M/R) nanorods upon W-doping are systematically studied. Moreover, the phase transition temperature (T_c_) of VO_2_ depending on oxygen nonstoichiometry is investigated in detail.

Vanadium dioxide (VO_2_) plays a crucial role in many fundamental research and practical applications. For instance, Mott field-effect transistor, light modulator and optical storage medium are potential products based on VO_2_^1^,^2^,^3^. Moreover, VO_2_ with a metal-insulator phase transition (MIT) is a key material for applying to thermochromic smart windows because it exhibits a reversible structural transformation from an infrared-transparent monoclinic phase (VO_2_(M_1_)) at low temperature to an infrared-reflective rutile state (VO_2_(R)) at higher temperature than the transition, while maintaining certain visible transmittance^4^,^5^,^6^,^7^. Whereas, VO_2_ exhibits hysteresis in its phase transition properties and mechanical degradation on passing through the MIT because of stresses during the structural change^8^. In addition, the phase transition temperature (T_c_) of MIT (68 °C) is always too high for the practical application of VO_2_-based materials^4^.

Nano-materials often exhibit extraordinary physical and chemical properties compared to their bulk counterparts^9^. One dimensional nanostructures, for example, nanorods represent particularly attractive because they present novel characteristics owing to their small radial dimension while retaining longitudinally connected substance^10^. In confined nanoscale system, more localized electronic states as well as narrower bands are usually supposed to increase the densities of states and lead to the superior phase transition behavior of VO_2_^11^. Using the hydration-cleavage-exfoliation solvothermal process, Banerjee and co-workers have reduced the T_c_ of undoped VO_2_ by synthesizing various sized nanostructures^12^. In their research, the phase transition temperature during the cooling cycle is more significantly affected than the heating cycle by nanostructuring, therefore, the hysteresis width is observed to be much wider for all the nanostructures. Gao and co-workers have also regulated the hysteresis width through the nano-size effect^13^, which provides a key that nanoscale VO_2_(M_1_/R) possesses the probability of tuning hysteresis width for obtaining a sharper, more reproducible phase transition. Up to now, more than 20 compounds of vanadium oxide (VO, V_2_O_3_, VO_2_, V_6_O_13_, V_8_O_15_, V_2_O_5_ and so on^14^) and 10 polymorphs of VO_2_ (B, A, T, M_1_, M_2_, R phase and so on^15^) had been reported. Only the VO_2_(M/R) (the M_1_ phase is referred to as the M phase of VO_2_ in this study) experiences a fully reversible MIT at the vicinity of room temperature (RT). Moreover, low temperature synthetic method has usually generated VO_2_(B) nanobelts and subsequently can be transformed to VO_2_(M/R) by the post-heating treatment, but the nanostructure has been nearly destroyed^16^,^17^,^18^. So it should be a challenge to synthesize pure phase VO_2_(M/R) with a shape controlled nanostructure.

The ongoing debate associated to the fundamental origin of the phase transition behavior in VO_2_ involves electron-correlation-driven (Mott transition)^19^,^20^, structure-driven (Peierls transition)^21^,^22^, or the cooperation of both^23^. W doping is known as an effective route to regulate electron density in the conduction band for decreasing T_c_ by approx. 20–26 °C/at% W for the bulk and by 50–80 °C/at% W in nanostructures^24^,^25^,^26^,^27^. Synthesis of VO_2_(M/R) by controlling both the shape of nanostructures and the amount of W dopant could be a good strategy to narrow the hysteresis width while reducing T_c_ for obtaining an excellent phase transition property of VO_2_-based materials. Of note, systematically experimental investigation of nonstoichiometric effect in VO_2_ has been insufficient. The phase transition behavior has been demonstrated to be also sensitive to vanadium or oxygen related vacancies, even a deviation in the oxygen stoichiometry by a few percent can cause the lattice structure change and result in several orders of magnitude difference in the resistivity transition or the phase transition temperature shift^28^,^29^. Therefore, studying on oxygen nonstoichiometry induced reduction of T_c_ will contribute to the general understanding of the intrinsic MIT mechanism in VO_2_.

In this study, we successfully explored a one-pot hydrothermal method to prepare VO_2_(M/R) with desired morphology. It is inspiring to discover that the W dopant promotes the generation of pure phase VO_2_(M/R) nanorods with high aspect ratio. Moreover, the effect of oxygen nonstoichiometry on the structural phase transition and subsequently T_c_ of VO_2_ is discussed in detail.

## Results

### Shape-controlled synthesis and phase metamorphosis behavior upon W doping

**Figure 1:**
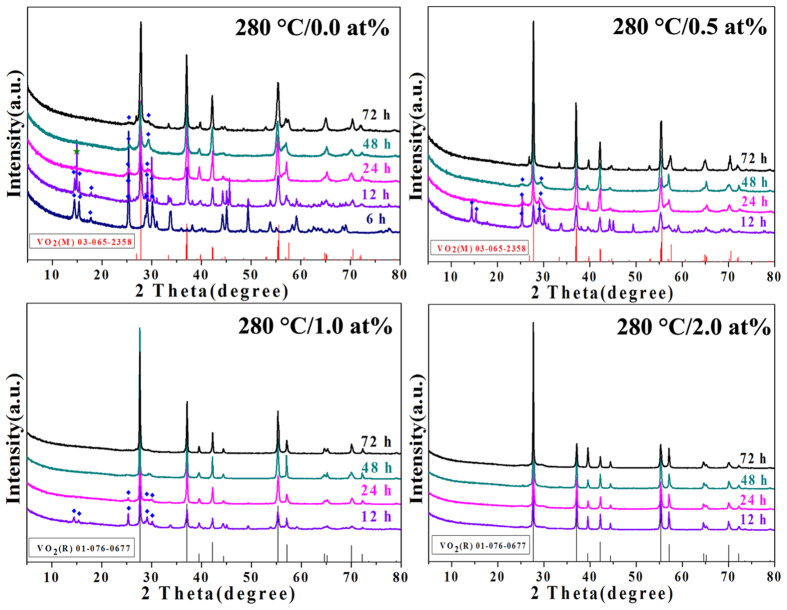
XRD patterns of W_x_V_1−x_O_2_ prepared at 280 °C with W doping levels ranging from 0.0 to 2.0 at% for different duration of hydrothermal time. The filled dark blue diamond and olive star are characteristic peaks for B and A phase of VO_2_ respectively. The red and black columns belong to standard pattern in JCPDS card No. 65–2358 for VO_2_(M) and No. 76–0677 for VO_2_(R) respectively.

**Figure 2:**
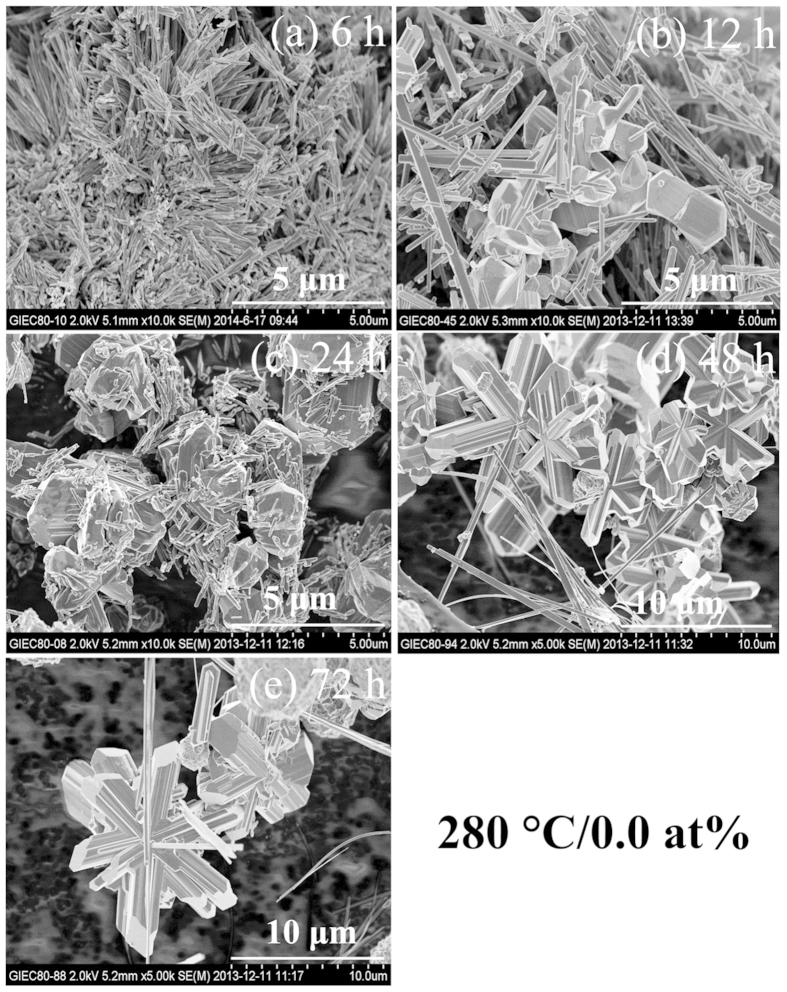
SEM images of undoped VO_2_ synthesized at 280 °C for different duration of hydrothermal time.

Figure 1 shows the crystalline phase metamorphic behavior of W_x_V_1−x_O_2_ with x = 0, 0.5, 1.0 and 2.0 at% respectively where the temperature being kept at 280 °C but the different duration of the hydrothermal time being applied. For the undoped VO_2_, pure phase VO_2_(B) is obtained for the duration of the hydrothermal time for 6 h. By increasing the duration of the hydrothermal time from 12 to 72 h, the peak of {011} for VO_2_(M) (M {011} at around 27.8°) appears and becomes more significant. However, there always exists the secondary phase VO_2_(B) in the final product. Serial SEM images in Fig. 2 show the morphology transition behavior of the undoped VO_2_ upon increasing the duration of the hydrothermal time. Products of the metastable VO_2_(B) are the tangled nanobelts in the morphology for the 6 h-sample. By increasing the duration of the hydrothermal time from 12 to 72 h, VO_2_(B) nanobelts always exist as partial morphology except the block or snowflake VO_2_(M). In conclusion, we could not synthesize pure phase VO_2_(M) without W doping.

**Figure 3:**
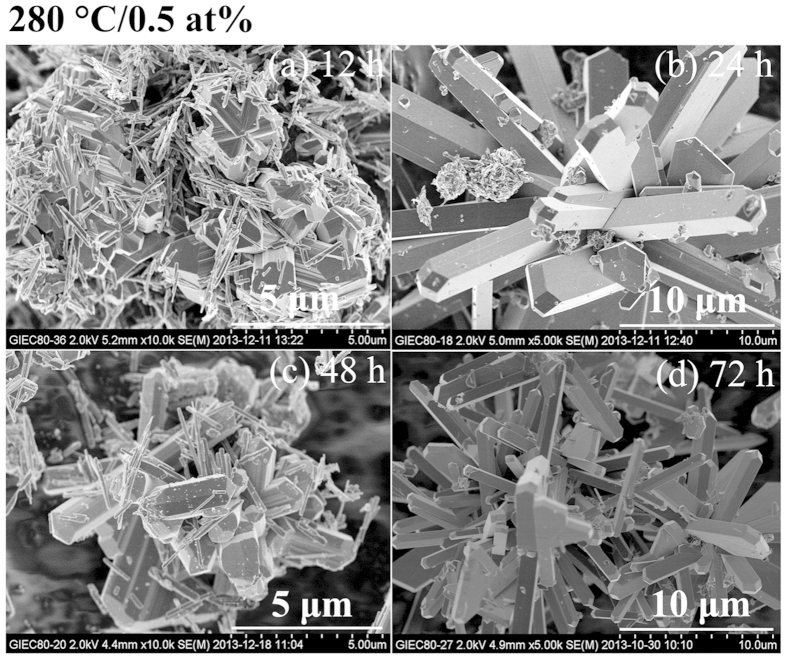
SEM images of W_x_V_1−x_O_2_ (x = 0.5 at%) synthesized at 280 °C for different duration of hydrothermal time.

**Figure 4:**
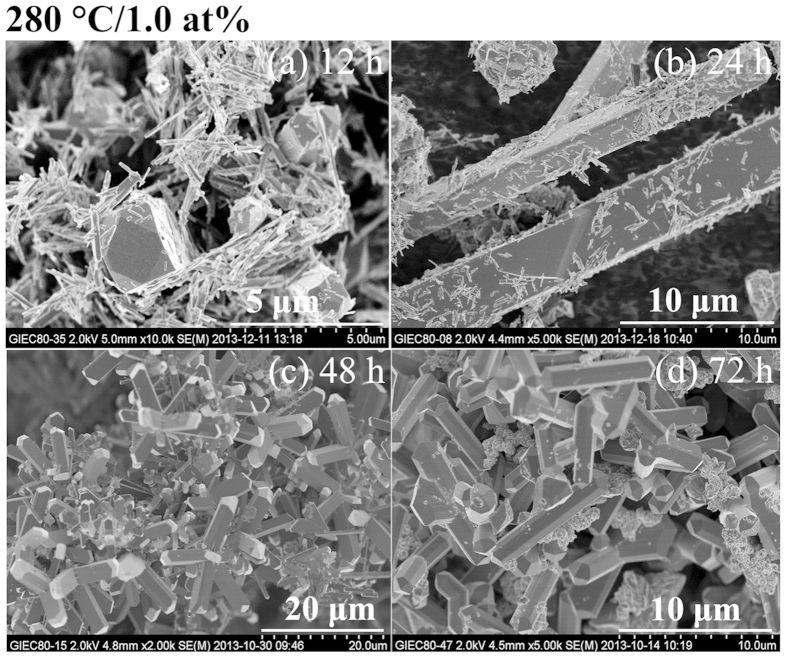
SEM images of W_x_V_1−x_O_2_ (x = 1.0 at%) synthesized at 280 °C for different duration of hydrothermal time.

**Figure 5:**
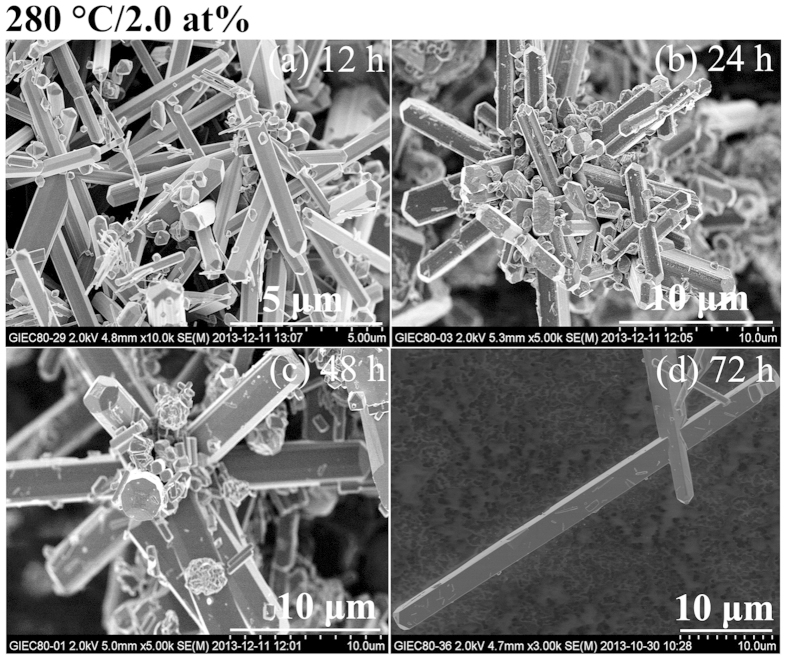
SEM images of W_x_V_1−x_O_2_ (x = 2.0 at%) synthesized at 280 °C for different duration of hydrothermal time.

**Figure 6:**
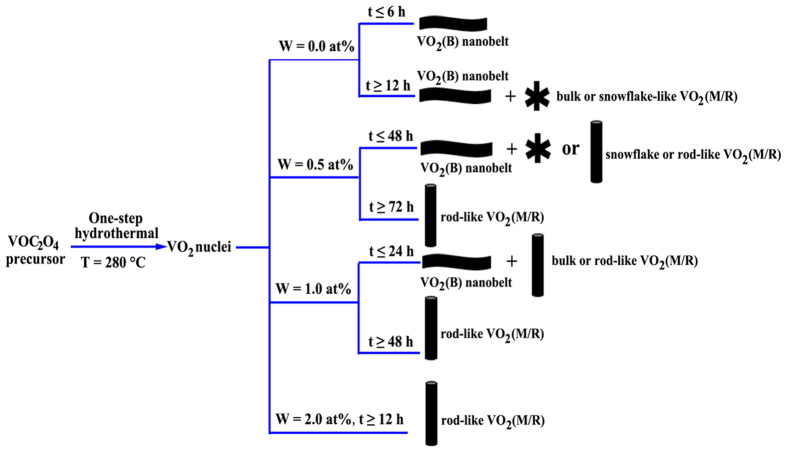
Schematic illustration of the morphology metamorphic behavior of VO_2_.

By increasing the W-doped level to 0.5 at%, VO_2_(B) nanobelts always exist as partial morphology except the snowflake or rod-like VO_2_(M) for the duration of the hydrothermal time ≤48 h as shown in Figs 1 and 3. Inspiringly, the peaks of VO_2_(B) vanish and pure phase VO_2_(M) (T_c _> RT) with uniformly rod-like morphology is successfully synthesized for the 72 h-sample. Meanwhile, for the hydrothermal samples prepared at 280 °C with W-doped of 1.0 and 2.0 at%, pure phase VO_2_(R) with uniformly rod-like morphology is obtained when the duration of the hydrothermal time ≥48 h and ≥12 h respectively as shown in Figs 1,4 and 5. The synchrotron radiation X-ray powder diffraction (SRXPD, λ = 0.50 Å) data confirm the pure phase VO_2_(M/R) is exactly free from the existing of the other V-O compounds and other VO_2_ phases, which was reported by our group in the recently study^30^. As an overall comparison, the schematic illustration of the morphology metamorphic behavior of VO_2_ is summarized in Fig. 6.

**Figure 7:**
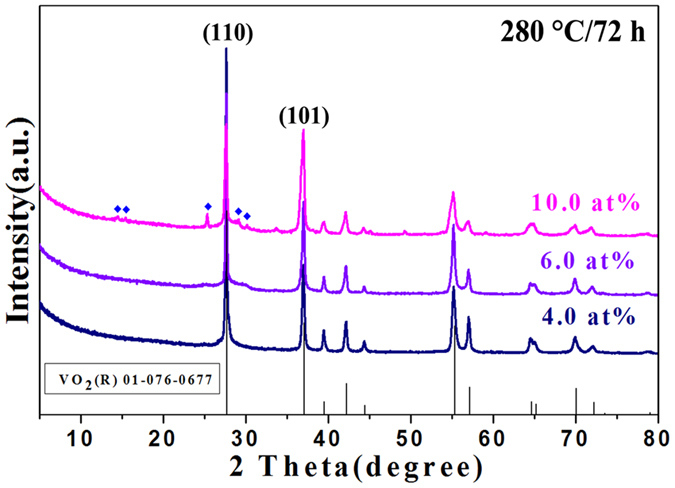
XRD patterns of the W_x_V_1−x_O_2_ prepared at 280 °C for 72 h with W doping levels ranging from 4.0 to 10.0 at%. The filled dark blue diamond is characteristic peaks for B phase of VO_2_. The black column belongs to standard pattern in JCPDS card No. 76–0677 for VO_2_(R).

**Figure 8:**
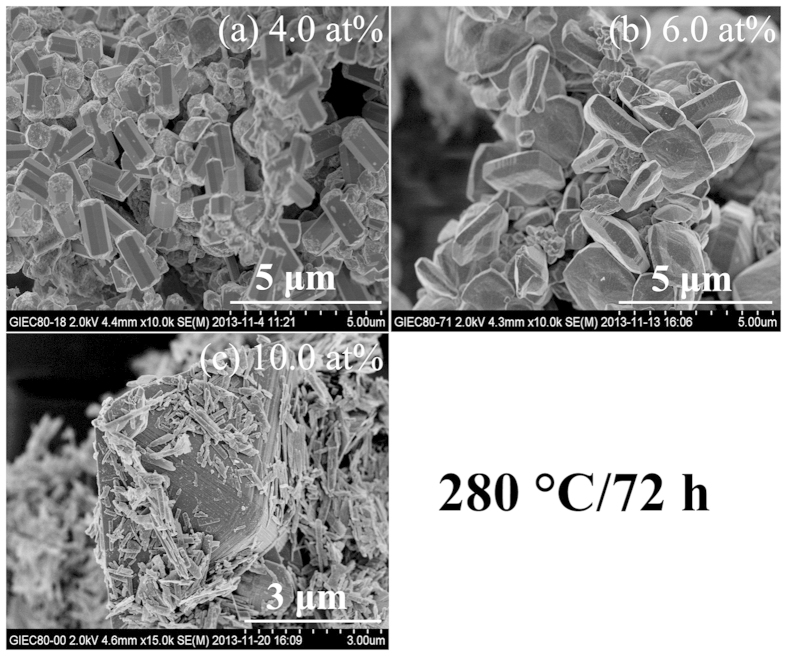
SEM images of the W_x_V_1−x_O_2_ prepared at 280 °C for 72 h with different W doping levels. (**a**) 4.0 at.% (**b**) 6.0 at.% (**c**) 10.0 at.%.

W_x_V_1−x_O_2_ with x = 4.0, 6.0 and 10.0 at% were prepared at 280 °C for 72 h to investigate more W dopant on the crystalline phase metamorphic and morphology transition behavior of the as-obtained products as shown in Figs 7 and 8. Pure phase VO_2_(R) is still obtained for the 4.0 and 6.0 at% sample. When the W-doped level increases to 10.0 at%, VO_2_(B) nanobelts are grown again besides the main phase VO_2_(R). The results indicate a certain doping level of W could promote VO_2_(B) metamorphoses to pure phase VO_2_(M/R), which agrees with the previous reports^14^,^18^. Whereas, more excess W dopant would prevent the metamorphosis from VO_2_(B) into VO_2_(R) thoroughly. The intensity ratio between the XRD peaks of {110} and that of {101} of VO_2_(R) prepared at 280 °C for 72 h with different W-doped levels is listed in Table 1. It increases significantly from 2.1 to 5.7 with increasing the W-doped level from 1.0 to 2.0 at%. The strong intensity of the {110} reflections points to the strongly preferential growth direction of the structures, as has also been noted previously for VO_2_ nanowires prepared at high temperatures by vapor transport^31^,^32^,^33^. Simultaneously, the aspect ratio of the VO_2_(R) nanorods increases from nearly 5 to 10 with the increased dopant. Whereas, if the W-doped level increases from 4.0 to 10.0 at%, the intensity ratio {110}/{101} decreases from 2.5 to 1.1. Meanwhile, the aspect ratio of nanorods decreases with the increased W dopant as shown in Fig. 8. Finally, the bulk crystal of VO_2_(R) is grown for the 10.0 at% sample. The results indicate a certain doping level of W can promote the preferential growth of R {110} and the increased aspect ratio of VO_2_(R) nanorods, whereas the excess W would restrain.

### The shape-controlled mechanism revealed by TEM

**Figure 9:**
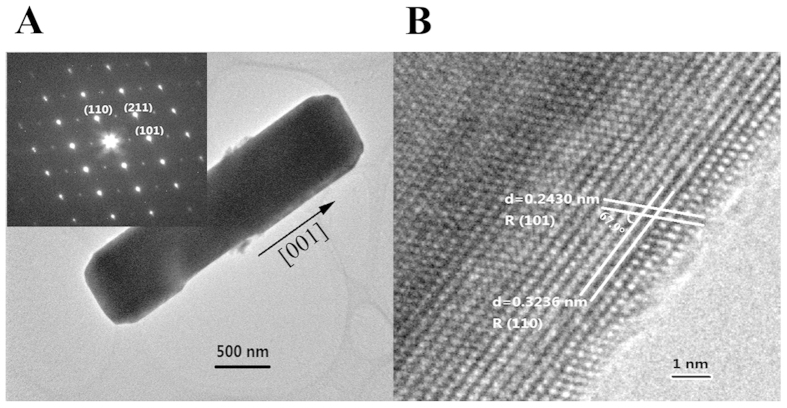
(**A**) TEM image of the single VO_2_ nanorod for the W-doped 4.0 at% sample and the corresponding SAED pattern (inset). (**B**) Lattice-resolved HRTEM image of the single nanorod.

The length of W-doped 4.0 at% VO_2_ nanorods (synthesized at 280 °C for 72 h) is about 2.5 μm with 600 nm in diameter as shown in the low magnification TEM image in Fig. 9A. The single-crystalline nanorods is confirmed by the lattice images of HRTEM and the inset SAED pattern as shown in Fig. 9. The lattice constants observed in Fig. 9B are 0.3236 and 0.2430 nm respectively, which can be indexed to the spacing of R {110} and R {101}, and the angle between the two lattice images is 67.9° in arc and this corresponds to the angle between the designated crystal planes of R (110) and R (101). In addition, the (001) plane orientation is just perpendicular to the nanorod' growth direction R (110), and revealing the preferential growth direction of the VO_2_(R) nanorods is along [001]. The results demonstrate that the preferential growth of nanorod' growth direction R (110) is responsible for the increased aspect ratio of VO_2_(R) nanorods. It is generally known that the greater the d-spacing, the atom arrange more closely on the crystal plane. For the body-centered tetragonal VO_2_(R), (110) with the largest d-spacing contributes to the lowest surface energy for the preferential growth of VO_2_ grains. According to the SAED pattern shown in the inset of Fig. 9A, the bright diffraction spots reveal the good crystallinity of the sample. Based on the Bragg equation, the diffraction spots can be ascribed to different crystal planes of VO_2_(R). The three Bravais lattice points shown in the SAED of the inset of Fig. 9A correspond to crystal planes of R (110), R (101) and R (211) respectively as indexed therein. This definitely demonstrates the nanorods belong to VO_2_(R). Moreover, no fringe spacing belongs to tungsten oxides or their derivatives are detected by HRTEM, which confirms the capture of W atoms into the crystal lattice of VO_2_ as mother matrix and the formation of homogeneous solid-solution of W_x_V_1−x_O_2_.

### Influence of oxygen nonstoichiometry on the phase transition behavior

**Figure 10:**
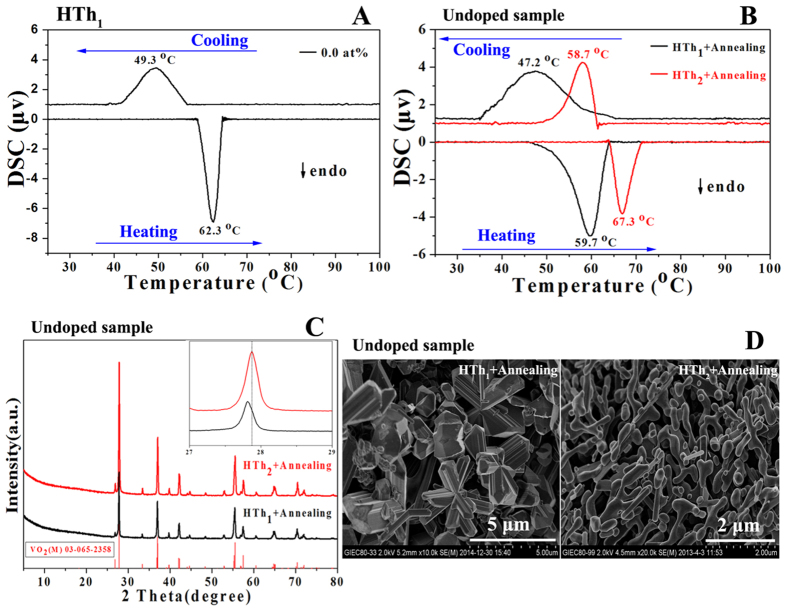
(**A**) DSC curve of the hydrothermal sample treated at 280 °C for 72 h (HTh_1_) with W-doped at 0.0 at%. (**B**) DSC curves for the undoped samples synthesized by the (HTh_1_ + Annealing) (annealing at 500 °C for 1 h in furnace) method and the (HTh_2_ + Annealing) (hydrothermal treated at 160 °C for 72 h) process respectively. (**C**) XRD patterns of the undoped samples synthesized by the designated two fabrication processes. The red column belongs to standard pattern in JCPDS card No. 65–2358 for VO_2_(M). (**D**) SEM images of the undoped samples synthesized by the designated two fabrication processes.

Figure 10A shows the DSC curve of the hydrothermally undoped sample treated at 280 °C for 72 h (HTh_1_). The endothermic and exothermal transition temperature is 62.3 and 49.3 °C during heating and cooling cycles respectively. Thus, the phase transition temperature (defined as T_c_ = (T_c,h_ + T_c,c_)/2) of the undoped micron-sized block and snowflake-like sample is about 55.8 °C, which is much lower than the transition temperature of undoped bulk VO_2_ (about 68 °C) reported by Morin^4^ and undoped nanobelts VO_2_ (64 °C) reported by Whittaker^24^. The hysteresis width (ΔT = T_c,h_−T_c,c_) of the undoped sample is about 13.0 °C.

To study the unusual low T_c_ for the HTh_1_ synthesized undoped sample, we directly compare the DSC for this sample by the after annealing (HTh_1 _+ Annealing) with that for the hydrothermal undoped one treated at 160 °C for 72 h and after annealing (HTh_2_ + Annealing) (annealing at 500 °C for 1 h in high-purity argon and this being also prepared by our group^34^) as shown in Fig. 10B. For the sake of comparison, we list Table 2 to show T_c_ and hysteresis width depending on the W-doped level and fabrication processes. When the undoped sample is synthesized by the (HTh_1_ + Annealing) process, T_c,h_ and T_c,c_ is about 59.7 and 47.2 °C respectively. Therefore, the T_c_ is about 53.5 °C, which is also lower than the (HTh_2_ + Annealing) fabricated undoped one (T_c_ being c.a 63.0 °C as shown in Table 2). Figure 10C shows the XRD patterns of the undoped samples synthesized by the designated two fabrication processes. The peaks of VO_2_(B) vanish and all of the peaks can be indexed to pure phase VO_2_(M) for the HTh_1_ synthesized undoped sample by the after annealing, which is similar to the (HTh_2 _+ Annealing) fabricated one. The inset close-up shows that M (011) peak shifts to low angles when comparing the (HTh_1_ + Annealing) synthesized undoped sample with those by the (HTh_2 _+ Annealing) synthesized one, which indicates the lattice spacing of M (011) increases. Both micron-sized snowflake and block-like morphologies are observed for the (HTh_1 _+ Annealing) synthesized undoped sample as shown in Fig. 10D. Whereas, nanostructure is grown by the (HTh_2_ + Annealing) fabrication process. Thanks to the formation energies of oxygen vacancies in rutile oxides are very high, the high hydrothermal temperature (280 °C) and reductive hydrothermal atmosphere for the (HTh_1_ + Annealing) method may contribute to the generation of oxygen vacancies to form nonstoichiometric VO_2-δ_ compared to the (HTh_2_ + Annealing) process (160 °C), and this would promote the lattice structural transition^35^,^36^.

### Discussion

**Figure 11:**
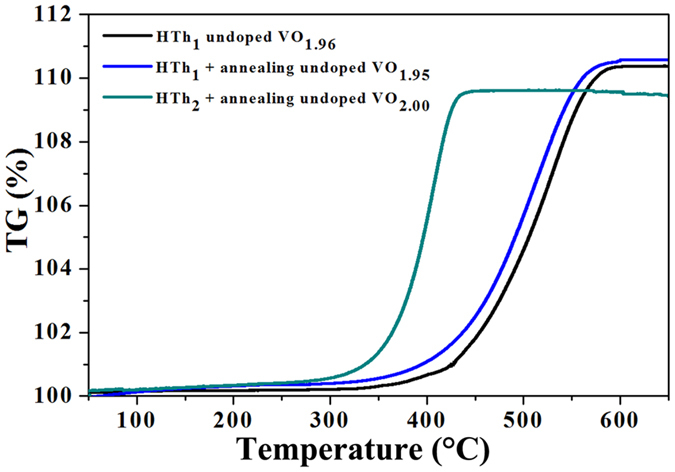
The thermogravimetric analysis of the samples.

To determine the oxygen stoichiometry, the thermogravimetric analysis of the samples was conducted as shown in Fig. 11. According to the TG curves, it can be found there exists one stage for the complete oxidization of the samples in the range of 300–600 °C. The weight gain (Δ_TG_) is about 10.4 %, 10.5 % and 9.6 % for the HTh_1_ synthesized undoped VO_x_, (HTh_1_ + annealing) undoped VO_y_ and (HTh_2_ + annealing) undoped VO_z_ respectively. The reaction equations for the oxidization of the samples can be given as follows (1):




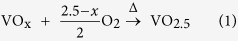







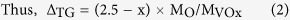




Where M_O_ and M_VOx_ represent molar mass of oxygen and VO_x_ respectively. When combining the above formulas (2) and experimental results, we can work out x = 1.96, y = 1.95 and z = 2.00 respectively. The fact demonstrates that oxygen deficiency is formed in the HTh_1_ synthesized undoped VO_1.96_ and (HTh_1_ + annealing) undoped VO_1.95_, and the precisely stoichiometric VO_2.00_ is formed in the (HTh_2_ + annealing) undoped sample. Son and co-workers have synthesized monoclinic VO_2_ micro- and nanocrystals by optimizing the hydrothermal conditions^37^. In their research, the phase transition temperature of stoichiometric VO_2.00_ microrods is around 68 °C. Usually, the T_c_ of MIT for VO_2_ is affected by doping, nanoscaling, nonstoichiometry, strain and etc^12^,^30^,^38^,^39^. For the HTh_1_ synthesized undoped micron-sized VO_1.96_, the reason for the unusual low T_c_ may be due to the oxygen nonstoichiometry. The nano-size effect may be responsible for the relative lower T_c_ (63 °C) of the (HTh_2_ + Annealing) synthesized stoichiometric VO_2.00_ nanostructure.

**Figure 12:**
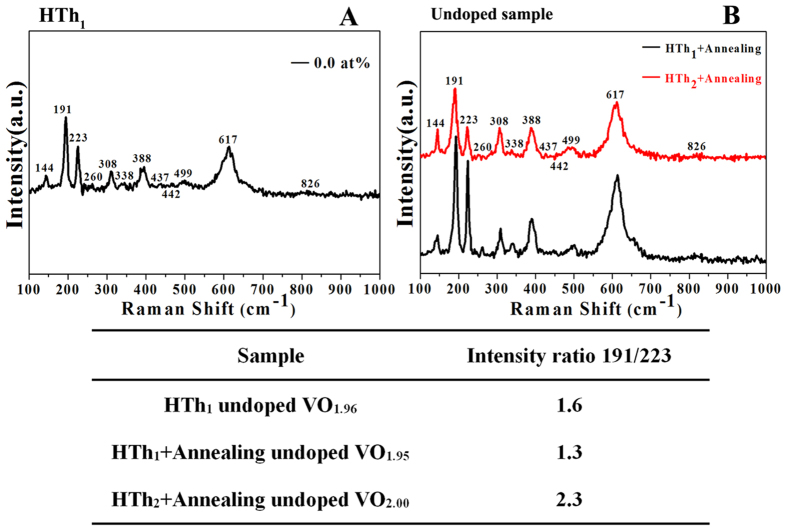
(**A**) Raman spectra of the HTh_1_ synthesized sample with W-doped at 0.0 at% in the Raman shift range 100–1000 cm^−1^. (**B**) Raman curves for the undoped samples synthesized by the (HTh_1 _+ Annealing) method and the (HTh_2 _+ Annealing) process respectively.

Figure 12A,B shows the Raman spectra of the samples depending on dopant level and fabrication processes. The peaks in the Raman spectra are all identified as 144 (B_1g_), 191 (A_g_), 223 (A_g_), 260 (A_g_), 308 (A_g_), 338 (A_g_), 388 (A_g_), 437 (A_g_), 442 (E_g_), 499 (A_g_), 617 (A_1g_), and 826 (B_2g_) cm^−1^ respectively, and these Raman-active modes are the clear evidence of the existing of VO_2_(M) belonging to space group 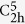, which agrees with the identified Raman peaks by other researchers^40^,^41^,^42^,^43^. The intensity ratio between the peak of 191 and that of 223 cm^−1^ (191/223) of the HTh_1_ synthesized undoped VO_1.96_ is 1.6. When comparing the (HTh_2 _+ Annealing) synthesized undoped VO_2.00_ with those by the (HTh_1 _+ Annealing) synthesized undoped VO_1.95_, the intensity ratio decreases from 2.3 to 1.3. H. T. Kim and co-workers have studied Raman spectra for the MIT of the undoped VO_2_ in detail and deduced the conclusion that the Raman-active A_g_ modes at 191 and 223 cm^−1^ were explained by the pairing and the tilting of V cations respectively^43^. Hence, the decreased relative intensity of 191 cm^−1^ peak suggests the depairing of V cations and the occurring of the localized structural phase transition (SPT, induced possibly by oxygen nonstoichiometry for the HTh_1_ synthesized undoped VO_1.96_ and (HTh_1_ + annealing) undoped VO_1.95_), and this might cause the transformation from the intrinsic structure of the matrix of VO_2_(M) to the localized rutile structure. In addition, the local rutile structure is the structure-guided domain, which will act as the initial nucleation site for the whole SPT^44^. This process might promote MIT for the origin of the lowering T_c_. However, this origin is still under the debate among the concerned experts as cited in the literature by Y. Xie *et al.* for an example, who pointed out that the atomic structure of isolated W dopant play a role in driving the nearby symmetric monoclinic VO_2_ lattice towards rutile phase, resulting in the depression of T_c_^45^,^46^. Hence the exact mechanism for the observed unusual phenomena requires our further investigation.

## Conclusions

In this study, pure phase VO_2_(M/R) with controlled morphology were successfully prepared via one-step hydrothermal method. The addition of a certain level of W (0.5–2.0 at%) is vital to synthesize the pure phase VO_2_(M/R) nanorods. The assured level of W doping can promote the preferential growth of {110} to form VO_2_(M/R) nanorods with high aspect ratio. It must be emphasized that the unusual low T_c_ equals to 55.8 and 53.5 °C is observed for the nonstoichiometric VO_1.96_ and VO_1.95_ in the bulk respectively, and the T_c_ is 63.0 °C for the precisely stoichiometric VO_2.00_ nanostructure. The present study demonstrates an improvement of the phase transition behavior and reduces the hindrances for the advanced applications of VO_2_-based materials.

## Methods

### Materials

Oxalic acid (H_2_C_2_O_4_·2H_2_O, AR) and vanadium pentoxide (V_2_O_5_, AR) were used as source material to prepare the vanadium precursor solution. Deionized water (ρ = 18.2 MΩ.cm) was used to prepare all aqueous solutions. Ammonium tungstate hydrate ((NH_4_)_5_H_5_[H_2_(WO_4_)_6_]·H_2_O, AR) was chosen as the W dopant. All of these reagents were used without further purification.

### The preparation process

The detail of this part has been described in previous report^30^. Briefly, V_2_O_5_ and oxalic acid (1: (1–3) in molar ratio) were directly added to 75 ml deionized water at RT. Then, a certain amount of W dopant was dispersed into the above solution with magnetic stirring. After mixing for 1 h, the resulting precursor was transferred into a 100 mL stainless steel autoclave with polyphenylene cup, then being sealed and maintained at 280 °C for 6–72 h. After the autoclave cooling to RT, a dark blue precipitate was obtained. The product was washed with deionized water and acetone for several times, then centrifuged at 8000 rpm for 8 min and dried in vacuum at 60 °C for 6 h.

In this study, (NH_4_)_5_H_5_[H_2_(WO_4_)_6_]·H_2_O was used as the W dopant, and the reported W-doped content here is based on the quantity of W atoms added in the feed. The sample synthesized by the duration of the hydrothermal time for 6 or 72 h is simplified to the 6 or 72 h-sample.

### Characterization techniques

The phase purity of the products was examined by an X-ray diffractometer (XRD, PANalytical X'pert Pro MPD) in the 2θ range of 5–80° with the step of 0.0083° using Cu-Kα radiation (λ = 1.54178 Å). The operating voltage and current were kept at 40 kV and 40 mA, respectively. The morphology and dimensions of the products were investigated using a field emission scanning electron microscope (FESEM, S-4800, Hitachi Japan) under the operating voltage of 2 kV. A JEOL-2100F instrument operated at 200 kV was used to acquire high-resolution transmission-electron-microscopy (HRTEM) images and selected area electron diffraction (SAED) patterns. Raman scattering spectra of the samples were recorded on a LabRAM HR800 micro-Raman spectrometer using a 532 nm wavelength YAG laser. The phase transition properties depending on the surrounding temperature of the as-prepared VO_2_ were studied by differential scanning calorimetry (HDSC, PT500LT/1600) under the temperature range from 25 to 100 °C under the circulatory heating/cooling cycles. The thermogravimetric analysis (TG) of the samples was conducted on a Nicolet 6700-Q50 thermal analyzer under dry air flow in the range of 50–650 °C with a heating rate of 5 °C min^−1^.

## Additional Information

**How to cite this article**: Chen, R. *et al.* Shape-controlled synthesis and influence of W doping and oxygen nonstoichiometry on the phase transition of VO_2_. *Sci. Rep.* **5**, 14087; doi: 10.1038/srep14087 (2015).

## Figures and Tables

**Table 1:** Intensity ratio between the XRD peaks of {110} and that of {101} of W_x_V_1−x_O_2_ prepared at 280 °C for 72 h with different W-doped levels.

**W-doped level (at. %)**	**Intensity ratio {110}/{101}**
1.0	2.1
2.0	5.7
4.0	2.5
6.0	1.9
10.0	1.1

**Table 2:** DSC parameters of the HTh_1_ synthesized sample with W-doped at 0.0 at% and of the undoped samples synthesized by the (HTh_1_ + Annealing) method and the (HTh_2 _+ Annealing) process respectively.

**Sample**	**Phase transition temperature**		Hysterisis width△T	**T_c_**
	Heating cycleT_c,h_	Cooling cycleT_c,c_		
HTh_1_ undoped VO_1.96_	62.3 °C	49.3 °C	13.0 °C	55.8 °C
HTh_1 _+ Annealing undoped VO_1.95_	59.7 °C	47.2 °C	12.5 °C	53.5 °C
HTh_2 _+ Annealing undoped VO_2.00_	67.3 °C	58.7 °C	8.6 °C	63.0 °C
